# Thrombosis and Anticoagulation Therapy in Systemic Lupus Erythematosus

**DOI:** 10.1155/2022/3208037

**Published:** 2022-06-27

**Authors:** Wenjun Yuan, Fengjun Guan

**Affiliations:** Department of Pediatrics, Affiliated Hospital of Xuzhou Medical University, Xuzhou, Jiangsu 221002, China

## Abstract

Systemic lupus erythematosus (SLE) is an autoimmune inflammatory disease in which pathogenic autoantibodies and immune complexes are formed and mediate multiple organ and tissue damage. Thrombosis is one of the most common causes of death in patients with SLE. Anticoagulant therapy blocks the vicious cycle between inflammation and thrombosis, which may greatly improve the long-term prognosis of patients with SLE. However, the etiology and pathogenesis of this disease are very complicated and have not yet been fully clarified. Therefore, in the present review, we will highlight the characteristics and mechanisms of thrombosis and focus on the anticoagulant drugs commonly used in clinical practice, thus, providing a theoretical basis for scientific and reasonable anticoagulant therapy in clinical practice.

## 1. Introduction

Systemic lupus erythematosus (SLE) is a systemic autoimmune disease with vasculopathy, as demonstrated by varying clinical presentations ranging from mild mucocutaneous disorders to multiorgan involvement in patients. The global incidence rate of SLE ranges from 1.5 to 11 per 100,000 person years, while the prevalence ranges from 13 to 7,713.5 per 100,000 individuals [1]. The reasons for these differences may be due to actual variation and due to differences in study design and case definition. Women of childbearing age are usually vulnerable to this disease. Genetic factors, including polygenic and monogenic factors (such as HLA, IRF5, ITGAM, STAT4, and CTLA4) [2] and genetic interactions with environmental factors, particularly UV light exposure, the Epstein–Barr virus (EBV) infection [3], hormonal factors, smoking [4], or medications [5] (such as procainamide, hydralazine, quinidine, isoniazid, TNF-*α* inhibitors [6], and anticonvulsants [7, 8]) are associated with the pathogenesis of SLE. Thromboembolic diseases were responsible for one of every four deaths worldwide in 2010 and are the leading cause of death in patients with SLE [9]. SLE patients have 25-to 50-fold higher incidence of thrombosis than the general population [10], with an incidence of venous or arterial thrombosis exceeding 10%; the incidence rate exceeds 50% in high-risk patients [11]. Men are more likely to experience thrombotic events than women [12, 13], and previous studies have shown that the risk of myocardial infarction is 3-fold higher in men than in women [14]. Thrombosis is one of the most common causes of death in patients with SLE [15]. However, most studies have focused on patients with antiphospholipid syndrome (APS) or high-risk factors, ignoring that SLE itself is an independent risk factor for thrombotic events; moreover, anticoagulation therapy has also been mostly aimed at patients with APS and pregnant patients, and the need for preventive anticoagulation therapy for patients with SLE has been rarely studied. Therefore, in the present review, we will focus on the causes of thrombosis in SLE and the commonly used anticoagulant drugs in clinical practice.

### 1.1. Search Strategy

To identify all available studies, a detailed search pertaining to thrombosis and anticoagulation therapy in SLE was conducted. A systematic search was performed in the electronic database PubMed (NCBI) by using the following search terms in all possible combinations: systemic lupus erythematosus, autoimmune disease, arterial thrombosis, vein thrombosis, cardiovascular disease, anticoagulation, antithrombotic treatment, and antithrombotic prophylaxis. The last search was performed on March 15, 2022.

## 2. Thrombosis in SLE

Thrombosis is a pathological process that involves the formation of blood clots or emboli in blood vessels under certain conditions. A prospective 5-year follow-up study of 219 patients with SLE demonstrated that 16% of patients had a thrombotic event during the study period, among which 3.5% had arterial thrombosis and 12.5% had venous thrombosis [16]. Smoking, old age, disease activity, use of a lupus anticoagulant, and glucocorticoid dose were observed to be the risk factors for the occurrence of venous thrombosis in lupus patients [17], whereas diabetes mellitus, hypertension, dyslipidemia, nephrotic syndrome, and chronic damage were found to be associated with arterial thrombosis [10]. The etiology and pathogenesis of SLE are very complicated and have not yet been fully clarified. Recent studies have reported that the contributing factors to thrombosis in lupus are mainly related to major factors such as vascular endothelial injury caused by autoantibodies, neutrophil extracellular traps (NETs), scavenger receptors, protein C pathway disorders, and glucocorticoid treatment.

### 2.1. Vascular Endothelial Injury

Endothelial cells maintain the normal blood coagulation function through a dynamic balance between coagulation and anticoagulation [18]. In flares of SLE, the vascular endothelium plays a pivotal role in initiating vasculopathy and thus contributes to organ injury. Immune complexes, autoantibodies, and various cytokines (TNF-*α*, MCP-1, IL-6, IL-8, IL-17, IL-12, and IL-18 [19–21]) are responsible for vascular endothelial injury in lupus. This is described in greater detail in the following section.

#### 2.1.1. Anti-Endothelial Cell Antibody-Mediated Vascular Endothelial Injury

Anti-endothelial cell antibody (AECA) is an antibody that plays a role in SLE by acting as a potential trigger of vasculopathy; it belongs to immunoglobulin A, G, or M [22] and binds to antigens through the F (ab) 2 region [23]. AECA is a heterogeneous group of autoantibodies that can react with different endothelial cell-associated antigenic structures [24], such as heparin-like compounds, DNA and DNA-histone complexes, PO and L6 ribosome proteins, elongation factor 1a, fibronectin, and *β*2-glycoprotein I [25], which promote tissue factor (TF) production and lead to vascular damage. Endothelial cell apoptosis may also be induced by AECA [26]. Several studies have also revealed that the presence of AECA is associated with renal involvement, vascular lesions, pulmonary hypertension, anticardiolipin antibodies, and thrombosis in lupus [27–29]. Therefore, AECA is one of the important factors of vascular endothelial injury.

#### 2.1.2. Antiphospholipid Antibody-Mediated Vascular Endothelial Injury

Statistics indicate that 30% to 50% of patients with SLE are positive for antiphospholipid antibody (aPL). aPL is widely considered one of the top risk factors for thrombosis [30]. It is speculated that aPL interferes with the endothelial function and promotes thrombosis. Amital et al. suggested that the deposition of aPL in the heart valves initiates the inflammatory process. Supporting evidence for this hypothesis was provided by Afek et al., who demonstrated that the markers of endothelial cell activation were upregulated in the valves of patients with APS [31]. Manukyan et al. showed that aPL can induce the expression and procoagulant activity of TF in monocytes and endothelial cells. These results indicate that aPL plays an important role in TF expression. The high expression of TF increases the production of activated coagulation factors FVII, FX, and thrombin which contribute to the development of a hypercoagulable state and an increased risk of thrombosis. In conclusion, aPL induces the production of proadhesive, proinflammatory, and procoagulant molecules that provide a persuasive explanation for the induction of thrombosis in APS [31].

#### 2.1.3. Anti-Neutrophil Cytoplasmic Autoantibody-Mediated Vascular Endothelial Injury

Anti-neutrophil cytoplasmic autoantibodies (ANCA) are a class of autoantibodies responsible for causing systemic vascular inflammation by binding to target antigens on neutrophils [32]. Several studies have shown that ANCA can activate neutrophils that attach to the endothelium of the blood vessels and release reactive oxygen species (ROS), nitric oxide, inflammatory cytokines (TNF-*α*, IL-1*β*, IL-8, and IL-12), toxic substances (serine proteases), and NETs, which result in vascular endothelial injury in small blood vessels [33, 34].

#### 2.1.4. Coagulation System Activation

**Figure 1:**
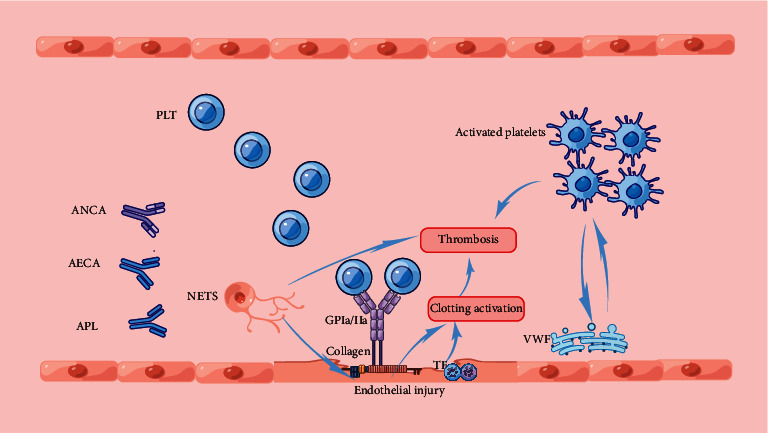
When endothelial cells are damaged by autoantibodies (ANCA,AECA,APL) and neutrophil extracellular traps, collagen and TF are exposed to the circulating blood, which the coagulation cascade is activated. Circulating platelets adhere directly to collagen via glycoprotein Ia/IIa surface receptors. This adhesion is further enhanced by the release of von Willebrand factor (vWF) from the damaged vascular endothelium and activated platelets. These interactions further activate platelets,which ultimately lead to increased platelet aggregation and thrombosis.

Endothelial cells provide a nonthrombotic surface under physiological conditions, which avoids the adhesion of platelets or other blood cells and thus prevents the occurrence of clotting cascades. When endothelial cells are damaged by autoantibodies, a series of coagulation reactions are initiated [35]. If there is a vessel damage, vasoconstriction occurs as a critical initial response. This causes a reduction in vessel diameter and slows down the blood flow, which is the hemodynamic basis for the subsequent hypercoagulability processes [18, 36]. Circulating blood cells and endothelial cells lining blood vessels generally do not express TF and are exposed to blood after vascular injury [18, 37]. TF is a promoter of the extrinsic pathway [38]. Moreover, when the endothelium is damaged, the underlying collagen is exposed to circulating platelets, which activates the intrinsic coagulation pathway. Platelets that circulate in the bloodstream adhere directly to collagen through the glycoprotein (GP) Ia/IIa surface receptors. This adhesion is further enhanced by the von Willebrand factor (vWF) released by vascular endothelial cells and platelets. These interactions also activate platelets. Activated platelets release ADP, serotonin, platelet activating factor (PAF), vWF, and thromboxane A2 (TXA2) into plasma, which subsequently activates additional platelets. Activated platelets change their shape from spherical to stellate, and fibrinogen is crosslinked with GP IIb/IIIa, which contributes to the aggregation of adjacent platelets [39]. Eventually, these reactions result in increased platelet aggregation and an increased risk of thrombosis (Figure 1).

### 2.2. NETs

Neutrophils are well known as an important part of innate immunity [40]. The role of neutrophils in thrombosis has only recently received attention. Darbousset et al. demonstrated that neutrophils are the first cytokines to be recruited to the location of endothelial dysfunction prior to thrombosis [41]. Neutrophils can cause pathological venous and arterial thrombosis or “immune thrombosis” by releasing NETs, which are networks of chromatin fibers released during neutrophil necrosis. NETs include histones, antimicrobial peptides, and oxidizing enzymes such as neutrophil elastase and myeloperoxidase [42]. Neutrophils have been shown to have an enhanced ability to produce NETs in patients with SLE [43]. Moreover, most patients with SLE have an impaired ability to degrade NETs [44]. Neutrophils that produce NET structures trigger an inflammatory response that leads to endothelial damage and induces dendritic cells to produce interferons, thus, amplifying the autoimmune response [45]. NETs play a crucial role in thrombosis by participating in platelet adhesion and fibrin generation [46]. Therefore, intervening NETs could be a potential target for anticoagulation therapy.

### 2.3. Administration of Glucocorticoids

Synthetic glucocorticoids are commonly used as the first-line treatment of lupus because of their strong anti-inflammatory effects and immunosuppressive properties. However, glucocorticoids may damage the homeostasis of the coagulation system and increase the risk of thrombosis in patients with lupus [47]. Glucocorticoids may aggravate coagulation abnormalities by releasing coagulation factors, inhibiting fibrinolysis, and aggravating endothelial injury [48]. Glucocorticoids can increase the levels of blood clotting factors FVII, FVIII, FXI, and PAI-1, thereby promoting coagulation and inhibiting fibrinolysis [47]. Glucocorticoids can also cause abnormal lipid metabolism, thus aggravating the existing hypercoagulable state [48]. Consequently, during chronic maintenance treatment, glucocorticoids should be minimized to less than 7.5 mg/day and withdrawn when possible. If possible, the tapering/discontinuation of glucocorticoids should be expedited by initiating treatment with appropriate immunomodulatory agents [49].

### 2.4. Macrophage Scavenger Receptors

Scavenger receptors (SRs) have a wide range of functions and are thought to be involved in complex events such as antigen presentation, lipid metabolism, phagocytosis, and apoptotic cell clearance [50]. SRs are structurally diverse and are categorized into seven classes (A–G) according to the multidomain structure of the individual members [51]. Current studies have confirmed that the dysfunction of SRs is involved in the pathogenesis of SLE [52, 53]. CD36 is a transmembrane glycoprotein of the class B SR family that has proatherogenic properties in SLE [54, 55]. In addition, by stimulating CD36 on the surface of platelets, oxLDL promotes platelet activation and renders a prothrombotic state [50, 56]. Reiss AB reported that SLE patient plasma markedly stimulated expression of CD36 message in a dose-dependent manner in THP-1 human monocytes [57]. Therefore, a strategy to prevent atherosclerosis by blocking CD36 activity may be an attractive target for pharmacological intervention.

### 2.5. Protein C Pathway

The protein C pathway is a natural anticoagulant that plays an important role in regulating coagulation and fibrinolysis and in preventing thrombosis [58]. The pathway includes thrombin, thrombomodulin, endothelial cell protein C receptor (EPCR), protein C, and protein S [59]. Disorders of the protein C pathway in SLE have received considerable attention in recent years. Antithrombomodulin antibodies interfere with the activated protein C (APC) and aPL interferes with the protein C pathway, which leads to an increased risk of thrombosis [60]. The lupus anticoagulant also increases resistance to APC [61]. Although APC resistance increases the risk of venous thrombosis, it remains unclear whether it increases the risk of arterial thrombosis [62]. Patients with SLE have been found to carry anti-PS autoantibodies that can form immune complexes which induce increased protein S clearance or interfere with the protein C-protein S system [63]. Thus, modulation of this pathway may be therapeutically beneficial.

## 3. Current Treatment Options

Over the past decades, the treatment of SLE has shifted from the use of hydroxychloroquine (HCQ), glucocorticosteroids, and conventional immunosuppressive drugs to biological agents, among which belimumab is the first and only biological agent approved for treating SLE to date [64]. Because of the application of biological agents, the prognosis of patients with SLE has significantly improved; however, with the prolongation of patients' survival, the incidence of complications such as thrombosis has increased. Treatment strategies are mostly focused on controlling disease activity while minimizing the accumulation of damage associated with active disease and drug-related adverse effects [65, 66]; however, anticoagulant treatment strategies in lupus are limited. Therefore, we will focus on the commonly used anticoagulant drugs in clinical practice and their mechanism in treating SLE.

### 3.1. Aspirin

Aspirin is an acidic nonsteroidal drug with antipyretic, analgesic, and anti-inflammatory properties. Apart from its original use as an analgesic and antipyretic drug, aspirin is commonly used today in low doses to prevent cardiovascular disease (CVD) [67]. Aspirin induces an antithrombotic effect by suppressing platelet reactivity through the inhibition of the cyclooxygenase activity of prostanglandin H synthase-1 (COX-1), which inhibits TXA2 synthesis [68]. A long-term retrospective cohort study showed the use of low-dose aspirin as the primary prophylaxis for cardiovascular events in patients with SLE [69]. Multiple meta-analyses and EULAR recommended that low-dose aspirin reduces the risk of first thrombotic events in asymptomatic aPL individuals, patients with SLE, and pregnant women with APS [70–72]. Low doses of aspirin reduced the embryo resorption in a model of experimental APS induced in pregnant mice and restored placental hCG secretion abolished by the effect of aPL [73]. On the basis of these studies, it seems logical that all patients with SLE should be considered for low-dose aspirin treatment unless there are definite contraindications. The evidence, however, mainly comes from observational studies, and rigorous large-scale experimental studies are required to provide stronger scientific evidence in the future.

### 3.2. Warfarin

Warfarin is the most commonly used oral anticoagulant. It interferes with the formation of clotting factors II, VII, IX, and X, as well as proteins C and S by antagonizing vitamin K [74]. Warfarin is commonly used for thromboembolic prophylaxis in patients with APS [75, 76]. However, less evidence is available on the use of warfarin in patients with SLE. A prospective multicenter research trial showed that warfarin (1–5 mg/day) started at the same time as a steroid therapy for at least 3 months can prevent the occurrence of osteonecrosis associated with SLE [77]. The most common side effect is bleeding. Therefore, prothrombin time (PT) should be monitored during warfarin administration. To overcome the differences in PT acquisition through the use of different reagents, standardized ratios (international standardized ratios or INR) have been developed, with a target INR of 2.5 believed to provide a good balance between antithrombotic activity and bleeding risk. When PT and INR do not meet the standard levels, the drug should not be used [78]. Warfarin can cross the placenta, and exposure to warfarin between 6 and 12 weeks of gestation can cause fetal warfarin syndrome [79]. Therefore, the use of warfarin should be avoided during the 6–12 weeks of gestation [80].

### 3.3. Heparin

Heparin is a glycosaminoglycan that inhibits thrombin and several activated clotting factors (XIIa, IXa, XIa, and Xa) by inducing conformational alterations that enhance antithrombin III activity [81]. Currently, the commonly used heparins in clinical practice include unfractionated heparin and low-molecular-weight heparin. Because unfractionated heparin has a highly variable dose-response relationship, activated partial thromboplastin time (aPTT) levels need to be monitored frequently to ensure treatment levels [82]. Low-molecular-weight heparin also shows little nonspecific binding with plasma proteins and endothelial cells, thereby reducing the risk of severe bleeding, thrombocytopenia, and osteoporosis caused by heparin [83]. Therefore, low-molecular-weight heparin is often used as an adjuvant treatment for patients with SLE, which can effectively improve the pregnancy outcome of these patients [84, 85]. Girardiet al. conducted an experimental *in vivo* study and demonstrated that C3 and C5 activation can amplify the procoagulant effects of aPL. Heparin appears to prevent aPL-induced pregnancy loss by inhibiting C3 and C5 activation rather than its anticoagulant effect [86].

### 3.4. HCQ

HCQ is a hydroxylated analog of chloroquine that inhibits the plasmodium heme polymerase and was originally used as an antimalarial drug [87]. However, recent studies have revealed that it can not only block antigen presentation, reduce T cell activation [88], and inhibit the production of proinflammatory cytokines and angiogenesis [89] but it can also serve as a basic medicine for lupus. More importantly, HCQ possesses multiple hematological mechanisms, including reduction in red blood cell sludging, blood viscosity, and platelet aggregation, which may explain its benefit as an antithrombotic agent [90]. Several studies have shown that HCQ is protective against thrombosis [91–93]. HCQ can also inhibit the binding of the antiphospholipid antibody *β*2-glycoprotein I complex to the phospholipid bilayer, which reduces the risk of thrombosis in APS [94]. Given that HCQ has been shown to be beneficial in patients with lupus by delaying the onset of damage in general increasing long-term survival, it is recommended for all patients with lupus [95]. However, its long-term use may be rarely accompanied by some serious side effects, especially retinopathy. Hence, the risk of eye complications should be assessed regularly [87].

## 4. Conclusion

In conclusion, patients with SLE are prone to thrombotic events. Currently, the management of anticoagulation relies on experts' opinion, and consensus is lacking. Despite the lack of evidence from randomized controlled trials, given the broad spectrum of beneficial effects and the safety profile, HCQ is recommended for all lupus patients, and all patients with SLE should be considered for low-dose aspirin treatment. In the future, more evidence-based studies are required to delineate the patients who may benefit from anticoagulation, especially non-high-risk patients with SLE.

## Data Availability

The data that support the findings of this study are available from the corresponding author upon reasonable request.

## References

[1] Barber M. R. W., Drenkard C., Falasinnu T. (2021). Global epidemiology of systemic lupus erythematosus. *Nature Reviews Rheumatology*.

[2] Ghodke-Puranik Y., Niewold T. B. (2015). Immunogenetics of systemic lupus erythematosus: a comprehensive review. *Journal of Autoimmunity*.

[3] Nelson P., Rylance P., Roden D., Trela M., Tugnet N. (2014). Viruses as potential pathogenic agents in systemic lupus erythematosus. *Lupus*.

[4] Simard J., Costenbader K., Liang M., Karlson E., Mittleman M. (2009). Exposure to maternal smoking and incident SLE in a prospective cohort study. *Lupus*.

[5] Vaglio A., Grayson P. C., Fenaroli P. (2018). Drug-induced lupus: traditional and new concepts. *Autoimmunity Reviews*.

[6] Chang C., Gershwin M. E. (2011). Drug-induced lupus erythematosus: incidence, management and prevention. *Drug Safety*.

[7] Jadhav P., Kulkarni T., Jadhav J., Desai S., Baviskar R. (2021). Levetiracetam-induced systemic lupus erythematosus. *Journal of the Royal College of Physicians of Edinburgh*.

[8] Sinha A., Hegde A., Kinra P., Neema S., Singh A. R. (2021). Oxcarbazepine-induced systemic lupus erythematosus: a rare and serious adverse drug reaction to a common anticonvulsant. *Indian Journal of Pharmacology*.

[9] Wendelboe A. M., Raskob G. E. (2016). Global burden of thrombosis: epidemiologic aspects. *Circulation Research*.

[10] Hinojosa-Azaola A., Romero-Diaz J., Vargas-Ruiz A. G. (2016). Venous and arterial thrombotic events in systemic lupus erythematosus. *Journal of Rheumatology*.

[11] Sarabi Z. S., Chang E., Bobba R. (2005). Incidence rates of arterial and venous thrombosis after diagnosis of systemic lupus erythematosus. *Arthritis & Rheumatism*.

[12] Riveros Frutos A., Casas I., Rúa-Figueroa I. (2017). Systemic lupus erythematosus in Spanish males: a study of the Spanish Rheumatology Society Lupus Registry (RELESSER) cohort. *Lupus*.

[13] Tan T. C., Fang H., Magder L. S., Petri M. A. (2012). Differences between male and female systemic lupus erythematosus in a multiethnic population. *Journal of Rheumatology*.

[14] Vavlukis M., Pop-Gjorcevab D., Poposka L., Sandevska E., Kedev S. (2021). Myocardial infarction in systemic lupus erythematosus - the sex-specific risk profile. *Current Pharmaceutical Design*.

[15] Al-Homood I. A. (2012). Thrombosis in systemic lupus erythematosus: a review article. *ISRN Rheumatology*.

[16] Klein A., Molad Y. (2021). Hematological manifestations among patients with rheumatic diseases. *Acta Haematologica*.

[17] Calvo-Alen J., Toloza S. M., Fernández M. (2005). Systemic lupus erythematosus in a multiethnic US cohort (LUMINA). XXV. Smoking, older age, disease activity, lupus anticoagulant, and glucocorticoid dose as risk factors for the occurrence of venous thrombosis in lupus patients. *Arthritis & Rheumatism*.

[18] Neubauer K., Zieger B. (2021). Endothelial cells and coagulation. *Cell and Tissue Research*.

[19] Robinson E. S., Werth V. P. (2015). The role of cytokines in the pathogenesis of cutaneous lupus erythematosus. *Cytokine*.

[20] Ruchakorn N., Ngamjanyaporn P., Suangtamai T. (2019). Performance of cytokine models in predicting SLE activity. *Arthritis Research and Therapy*.

[21] Bazzan M., Vaccarino A., Marletto F. (2015). Systemic lupus erythematosus and thrombosis. *Thrombosis Journal*.

[22] Praprotnik S., Blank M., Meroni P. L., Rozman B., Eldor A., Shoenfeld Y. (2001). Classification of anti-endothelial cell antibodies into antibodies against microvascular and macrovascular endothelial cells: the pathogenic and diagnostic implications. *Arthritis & Rheumatism*.

[23] Cieślik P., Hrycek A., Kłuciński P. (2008). Vasculopathy and vasculitis in systemic lupus erythematosus. *Polskie Archiwum Medycyny Wewnetrznej*.

[24] Arends S. J., Damoiseaux J. G., Duijvestijn A. M., van Paassen P. (2013). “Immunoglobulin G anti-endothelial cell antibodies: inducers of endothelial cell apoptosis in pulmonary arterial hypertension?. *Clinical and Experimental Immunology*.

[25] Meroni P. L., Tincani A., Sepp N. (2003). Endothelium and the brain in CNS lupus. *Lupus*.

[26] Jamin C., Dugué C., Alard J. E. (2005). Induction of endothelial cell apoptosis by the binding of anti-endothelial cell antibodies to Hsp60 in vasculitis-associated systemic autoimmune diseases. *Arthritis & Rheumatism*.

[27] Perry G. J., Elston T., Khouri N. A., Chan T. M., Cameron J. S., Frampton G. (1993). Antiendothelial cell antibodies in lupus: correlations with renal injury and circulating markers of endothelial damage. *The Quaterly Journal of Medicine*.

[28] Yoshio T., Masuyama J., Sumiya M., Minota S., Kano S. (1994). Antiendothelial cell antibodies and their relation to pulmonary hypertension in systemic lupus erythematosus. *Journal of Rheumatology*.

[29] Hill M. B., Phipps J. L., Milford-Ward A., Greaves M., Hughes P. (1996). Further characterization of anti-endothelial cell antibodies in systemic lupus erythematosus by controlled immunoblotting. *British Journal of Rheumatology*.

[30] de Groot P. G., de Laat B. (2017). Mechanisms of thrombosis in systemic lupus erythematosus and antiphospholipid syndrome. *Best Practice & Research Clinical Rheumatology*.

[31] Tenedios F., Erkan D., Lockshin M. D. (2005). Cardiac involvement in the antiphospholipid syndrome. *Lupus*.

[32] Suwanchote S., Rachayon M., Rodsaward P. (2018). Anti-neutrophil cytoplasmic antibodies and their clinical significance. *Clinical Rheumatology*.

[33] Hutton H. L., Holdsworth S. R., Kitching A. R. (2017). ANCA-associated vasculitis: pathogenesis, models, and preclinical testing. *Seminars in Nephrology*.

[34] Xiao H., Hu P., Falk R. J., Jennette J. C. (2016). Overview of the pathogenesis of ANCA-associated vasculitis. *Kidney Disease*.

[35] Wu K. K., Thiagarajan P. (1996). Role of endothelium in thrombosis and hemostasis. *Annual Review of Medicine*.

[36] Durand M. J., Gutterman D. D. (2013). Diversity in mechanisms of endothelium-dependent vasodilation in health and disease. *Microcirculation*.

[37] Smith S. A., Travers R. J., Morrissey J. H. (2015). How it all starts: Initiation of the clotting cascade. *Critical Reviews in Biochemistry and Molecular Biology*.

[38] Butenas S., Orfeo T., Mann K. G. (2009). Tissue factor in coagulation: which? where? when?. *Arteriosclerosis, Thrombosis, and Vascular Biology*.

[39] Coelho M. C., Santos C. V., Vieira Neto L., Gadelha M. R. (2015). Adverse effects of glucocorticoids: coagulopathy. *European Journal of Endocrinology*.

[40] Kubes P. (2018). The enigmatic neutrophil: what we do not know. *Cell and Tissue Research*.

[41] Darbousset R., Thomas G. M., Mezouar S. (2012). Tissue factor-positive neutrophils bind to injured endothelial wall and initiate thrombus formation. *Blood*.

[42] Kapoor S., Opneja A., Nayak L. (2018). The role of neutrophils in thrombosis. *Thrombosis Research*.

[43] Blanco L. P., Wang X., Carlucci P. M. (2021). RNA externalized by neutrophil extracellular traps promotes inflammatory pathways in endothelial cells. *Arthritis & Rheumatology*.

[44] Lee K. H., Kronbichler A., Park D. D.-Y. (2017). Neutrophil extracellular traps (NETs) in autoimmune diseases: a comprehensive review. *Autoimmunity Reviews*.

[45] O’Neil L. J., Kaplan M. J., Carmona-Rivera C. (2019). The role of neutrophils and neutrophil extracellular traps in vascular damage in systemic lupus erythematosus. *Journal of Clinical Medicine*.

[46] Carminita E., Crescence L., Panicot-Dubois L., Dubois C. (2022). Role of neutrophils and NETs in animal models of thrombosis. *International Journal of Molecular Sciences*.

[47] Isidori A. M., Minnetti M., Sbardella E., Graziadio C., Grossman A. B. (2015). Mechanisms in endocrinology: the spectrum of haemostatic abnormalities in glucocorticoid excess and defect. *European Journal of Endocrinology*.

[48] Vojnovic Milutinovic D., Teofilović A., Veličković N. (2021). Glucocorticoid signaling and lipid metabolism disturbances in the liver of rats treated with 5alpha-dihydrotestosterone in an animal model of polycystic ovary syndrome. *Endocrine*.

[49] Fanouriakis A., Kostopoulou M., Alunno A., Jayne D., Kouloumas M., Kuhn A. (2019). “Update of the EULAR recommendations for the management of systemic lupus erythematosus. *Annals of the Rheumatic Diseases*.

[50] Canton J., Neculai D., Grinstein S. (2013). Scavenger receptors in homeostasis and immunity. *Nature Reviews Immunology*.

[51] Ashraf M. Z., Sahu A. (2012). Scavenger receptors: a key player in cardiovascular diseases. *Biomolecular Concepts*.

[52] Wermeling F., Chen Y., Pikkarainen T. (2007). Class A scavenger receptors regulate tolerance against apoptotic cells, and autoantibodies against these receptors are predictive of systemic lupus. *Journal of Experimental Medicine*.

[53] Munoz L. E., Gaipl U. S., Franz S. (2005). SLE--a disease of clearance deficiency?. *Rheumatology*.

[54] Park Y. M. (2014). CD36, a scavenger receptor implicated in atherosclerosis. *Experimental & Molecular Medicine*.

[55] Tian K., Xu Y., Sahebkar A., Xu S. (2020). CD36 in atherosclerosis: Pathophysiological mechanisms and therapeutic implications. *Current Atherosclerosis Reports*.

[56] Silverstein R. L. (2009). Type 2 scavenger receptor CD36 in platelet activation: the role of hyperlipemia and oxidative stress. *Clinical Lipidology*.

[57] Reiss A. B., Wan D. W., Anwar K. (2009). Enhanced CD36 scavenger receptor expression in THP-1 human monocytes in the presence of lupus plasma: linking autoimmunity and atherosclerosis. *Experimental Biology and Medicine (Maywood)*.

[58] Costallat L. T., Ribeiro C. C., Annichino-Bizzacchi J. M. (1998). Antithrombin, protein S and protein C and antiphospholipid antibodies in systemic lupus erythematosus. *Sangre*.

[59] Esmon C. T. (2003). The protein C pathway. *Chest*.

[60] Urbanus R. T., de Laat B. (2010). Antiphospholipid antibodies and the protein C pathway. *Lupus*.

[61] Marciniak E., Romond E. H. (1989). Impaired catalytic function of activated protein C: a new in vitro manifestation of lupus anticoagulant. *Blood*.

[62] Vandenbroucke J. P., Koster T., Briët E., Reitsma P. H., Bertina R. M., Rosendaal F. R. (1994). Increased risk of venous thrombosis in oral-contraceptive users who are carriers of factor V Leiden mutation. *Lancet*.

[63] Guermazi S., Regnault V., Gorgi Y., Ayed K., Lecompte T., Dellagi K. (2000). Further evidence for the presence of anti-protein S autoantibodies in patients with systemic lupus erythematosus. *Blood Coagulation and Fibrinolysis*.

[64] Basta F., Fasola F., Triantafyllias K., Schwarting A. (2020). Systemic lupus erythematosus (SLE) therapy: the old and the new. *Rheumatology and Therapy*.

[65] Durcan L., O’Dwyer T., Petri M. (2019). Management strategies and future directions for systemic lupus erythematosus in adults. *Lancet*.

[66] Doria A., Gatto M., Zen M., Iaccarino L., Punzi L. (2014). Optimizing outcome in SLE: treating-to-target and definition of treatment goals. *Autoimmunity Reviews*.

[67] Ornelas A., Zacharias-Millward N., Menter D. G. (2017). Beyond COX-1: the effects of aspirin on platelet biology and potential mechanisms of chemoprevention. *Cancer Metastasis Review*.

[68] Kawai V. K., Avalos I., Oeser A. (2014). Suboptimal inhibition of platelet cyclooxygenase 1 by aspirin in systemic lupus erythematosus: association with metabolic syndrome. *Arthritis Care & Research*.

[69] Iudici M., Fasano S., Gabriele Falcone L. (2016). Low-dose aspirin as primary prophylaxis for cardiovascular events in systemic lupus erythematosus: a long-term retrospective cohort study. *Rheumatology*.

[70] Arnaud L., Mathian A., Devilliers H. (2015). Patient-level analysis of five international cohorts further confirms the efficacy of aspirin for the primary prevention of thrombosis in patients with antiphospholipid antibodies. *Autoimmunity Reviews*.

[71] Tektonidou M. G., Andreoli L., Limper M. (2019). EULAR recommendations for the management of antiphospholipid syndrome in adults. *Annals of the Rheumatic Diseases*.

[72] Arnaud L., Mathian A., Ruffatti A. (2014). Efficacy of aspirin for the primary prevention of thrombosis in patients with antiphospholipid antibodies: an international and collaborative meta-analysis. *Autoimmunity Reviews*.

[73] Cadavid A. P. (2017). Aspirin: the mechanism of action revisited in the context of pregnancy complications. *Frontiers in Immunology*.

[74] Mega J. L., Simon T. (2015). Pharmacology of antithrombotic drugs: an assessment of oral antiplatelet and anticoagulant treatments. *Lancet*.

[75] Kearon C., Akl E. A., Ornelas J. (2016). Antithrombotic therapy for VTE disease: CHEST guideline and expert panel report. *Chest*.

[76] Keeling D., Baglin T., Tait C. (2011). Guidelines on oral anticoagulation with warfarin - fourth edition. *British Journal of Haematology*.

[77] Nagasawa K., Tada Y., Koarada S. (2006). Prevention of steroid-induced osteonecrosis of femoral head in systemic lupus erythematosus by anti-coagulant. *Lupus*.

[78] Ruiz-Irastorza G., Khamashta M. A., Hughes G. R. (2001). Antiaggregant and anticoagulant therapy in systemic lupus erythematosus and Hughes’ syndrome. *Lupus*.

[79] Yurdakök M. (2012). Fetal and neonatal effects of anticoagulants used in pregnancy: a review. *Turkish Journal of Pediatrics*.

[80] Ruiz-Irastorza G., Crowther M., Branch W., Khamashta M. A. (2010). Antiphospholipid syndrome. *The Lancet*.

[81] Bussey H., Francis J. L. (2004). Heparin overview and issues. *Pharmacotherapy: The Journal of Human Pharmacology and Drug Therapy*.

[82] McRae H. L., Militello L., Refaai M. A. (2021). Updates in anticoagulation therapy monitoring. *Biomedicines*.

[83] Hirsh J., Warkentin T. E., Shaughnessy S. G. (2001). Heparin and low-molecular-weight heparin: mechanisms of action, pharmacokinetics, dosing, monitoring, efficacy, and safety. *Chest*.

[84] Mecacci F., Bianchi B., Pieralli A. (2009). Pregnancy outcome in systemic lupus erythematosus complicated by anti-phospholipid antibodies. *Rheumatology*.

[85] Ambrósio P., Lermann R., Cordeiro A., Borges A., Nogueira I., Serrano F. (2010). Lupus and pregnancy--15 years of experience in a tertiary center. *Clinical Reviews in Allergy and Immunology*.

[86] Girardi G., Redecha P., Salmon J. E. (2004). Heparin prevents antiphospholipid antibody-induced fetal loss by inhibiting complement activation. *Nature Medicine*.

[87] Ponticelli C., Moroni G. (2017). Hydroxychloroquine in systemic lupus erythematosus (SLE). *Expert Opinion on Drug Safety*.

[88] Goldman F. D., Gilman A. L., Hollenback C., Kato R. M., Premack B. A., Rawlings D. J. (2000). Hydroxychloroquine inhibits calcium signals in T cells: a new mechanism to explain its immunomodulatory properties. *Blood*.

[89] Wozniacka A., Lesiak A., Boncela J., Smolarczyk K., McCauliffe D. P., Sysa-Jedrzejowska A. (2008). The influence of antimalarial treatment on IL-1beta, IL-6 and TNF-alpha mRNA expression on UVB-irradiated skin in systemic lupus erythematosus. *British Journal of Dermatology*.

[90] Petri M. (2011). Use of hydroxychloroquine to prevent thrombosis in systemic lupus erythematosus and in antiphospholipid antibody-positive patients. *Current Rheumatology Reports*.

[91] Jung H., Bobba R., Su J. (2010). The protective effect of antimalarial drugs on thrombovascular events in systemic lupus erythematosus. *Arthritis & Rheumatism*.

[92] Belizna C. (2015). Hydroxychloroquine as an anti-thrombotic in antiphospholipid syndrome. *Autoimmunity Reviews*.

[93] Wang T.-F., Lim W. (2016). What is the role of hydroxychloroquine in reducing thrombotic risk in patients with antiphospholipid antibodies?. *Hematology American Society of Hematology Education Program*.

[94] Rand J. H., Wu X. X., Quinn A. S., Chen P. P., Hathcock J. J., Taatjes D. J. (2008). Hydroxychloroquine directly reduces the binding of antiphospholipid antibody-beta2-glycoprotein I complexes to phospholipid bilayers. *Blood*.

[95] Ruiz-Irastorza G., Ramos-Casals M., Brito-Zeron P., Khamashta M. A. (2010). Clinical efficacy and side effects of antimalarials in systemic lupus erythematosus: a systematic review. *Annals of the Rheumatic Diseases*.

